# Identification and Antimicrobial Activity of Medium-Sized and Short Peptides from Yellowfin Tuna (*Thunnus albacares*) Simulated Gastrointestinal Digestion

**DOI:** 10.3390/foods9091185

**Published:** 2020-08-27

**Authors:** Andrea Cerrato, Anna Laura Capriotti, Federico Capuano, Chiara Cavaliere, Angela Michela Immacolata Montone, Carmela Maria Montone, Susy Piovesana, Riccardo Zenezini Chiozzi, Aldo Laganà

**Affiliations:** 1 Department of Chemistry, Università di Roma “La Sapienza”, Piazzale Aldo Moro 5, 00185 Rome, Italy; andrea.cerrato@uniroma1.it (A.C.); annalaura.capriotti@uniroma1.it (A.L.C.); chiara.cavaliere@uniroma1.it (C.C.); susy.piovesana@uniroma1.it (S.P.); aldo.lagana@uniroma1.it (A.L.); 2 Istituto Zooprofilattico Sperimentale del Mezzogiorno, Via Salute 2, 80055 Portici (NA), Italy; federico.capuano@cert.izsmportici.it (F.C.); angela.montone@izsmportici.it (A.M.I.M.); 3 Department of Industrial Engineering, Università degli Studi di Salerno, Via Giovanni Paolo II 132, 84084 Fisciano (SA), Italy; 4 Biomolecular Mass Spectrometry and Proteomics, Bijvoet Center for Biomolecular Research, Utrecht Institute for Pharmaceutical Sciences, Utrecht University, Padualaan 8, 3584 CH Utrecht, The Netherlands; r.zenezinichiozzi@uu.nl; 5 CNR NANOTEC, Campus Ecotekne, University of Salento, Via Monteroni, 73100 Lecce, Italy

**Keywords:** bioactive peptides, simulated gastrointestinal digestion, short peptides, yellow fin tuna, antimicrobial activity, high resolution mass spectrometry

## Abstract

Due to the rapidly increasing resistance to conventional antibiotics, antimicrobial peptides are emerging as promising novel drug candidates. In this study, peptide fragments were obtained from yellowfin tuna muscle by simulated gastrointestinal digestion, and their antimicrobial activity towards Gram-positive and Gram-negative bacteria was investigated. In particular, the antimicrobial activity of both medium- and short-sized peptides was investigated by using two dedicated approaches. Medium-sized peptides were purified by solid phase extraction on C18, while short peptides were purified thanks to a graphitized carbon black sorbent. For medium-sized peptide characterization, a peptidomic strategy based on shotgun proteomics analysis was employed, and identification was achieved by matching protein sequence database by homology, as yellowfin tuna is a non-model organism, leading to the identification of 403 peptides. As for short peptide sequences, an untargeted suspect screening approach was carried out by means of an inclusion list presenting the exact mass to charge ratios (*m*/*z*) values for all di-, tri- and tetrapeptides. In total, 572 short sequences were identified thanks to a customized workflow dedicated to short peptide analysis implemented on Compound Discoverer software.

## 1. Introduction

Fish is considered the main food source of proteins worldwide; furthermore, fish proteins have an enormous potential as novel sources of bioactive peptides [1]. To date, several biological activities have been found in the hydrolysates of fish proteins, such as antihypertensive, antioxidant, antimicrobial, immunomodulatory, enhancement of mineral absorption, antithrombotic, antihypertensive, opioid and antioxidant activities and antiproliferative effects [2,3,4,5]. Among the countless biological activities already studied in muscle fish samples, antimicrobial activity is one of the most important, since fish antimicrobial peptides (AMPs) represent ancient host-defense effector molecules [6,7]. In fact, the fish immune system acts as the first line of defense in an aquatic environment, where there is a high level of risk of infection by pathogens, such as bacteria, viruses, parasites and fungi [8]. Recently, AMPs have been drawing attention as possible substitutes for conventional antibiotics, the resistance to which is continuously increasing [9]. AMPs possess amphipathic properties due to their positive charges and hydrophobic residues and have been proved to carry out an essential function for innate immunity against pathogenic organisms. The mechanisms of actions which AMPs exhibit are remarkably different from those of commonly used antibiotics, as they are able to fracture bacterial membranes, inhibit the biosynthesis of protein and DNA and other cellular processes, such as protein folding and metabolic turnover [10,11]. Thanks to their broad spectrum of mechanisms of action, AMPs show high antimicrobial activity in low concentrations against a broad spectrum of microorganisms, including Gram-positive and Gram-negative bacteria, fungi, and viruses [9]. Their identification and characterization are therefore of great importance for possible use in pharmaceutical and nutraceutical sectors.

Many studies have already demonstrated that a broad spectrum of AMPs is expressed by fish proteins, including defensins, cathelicidins, hepcidins, histone-derived peptides, and the fish-specific piscidins, which belong to the cecropin family [12]. It is widely recognized that AMPs are encrypted in parent proteins and they are in their inactive form before protein digestion [8]. For instance, AMPs can be produced after gastrointestinal digestion [13] or by the sequential use of proteinases such as pepsin, trypsin, alcalase or pancreatin and even by proteolytic microorganisms or enzymes during fermentation processes [14,15,16,17]. AMPs generally possess a minimum of 12 and a maximum of 50 amino acids in their sequences [13] and generally present cationic, amphipathic and α-helical characteristics, whereas some non-cationic AMPs have been reported [18].

Regarding small peptides (2–4 amino acids long), at present, only two studies reported the identification of two antimicrobial tripeptides (IQY and YVL) and two antimicrobial tetrapeptides (EIPT and CIRA) generated by the enzymatic hydrolysis of bovine lactoferrin and kappa casein. The small number of publications on short AMPs is mainly ascribed to the several issues in the separation and identification of small amino acidic sequences [19,20,21,22].

Given the above, the scope of this work was to develop a comprehensive methodology for the investigation of both short- and medium-sized AMPs in yellowfin tuna (*Thunnus albacares*) fillet samples. Simulated gastrointestinal digested peptides were obtained and fractionated in two fractions, one for medium-sized peptides and one specifically addressing the issue of the isolation and purification of short peptide sequences. Similarly, analysis and identification were considered as well, using a shotgun proteomics strategy for the identification of medium-sized peptides which included a protein sequence database search, and a suspect screening investigation specific for short peptides. The two extracts were subjected to an antimicrobial activity assay against the standard bacterial cultures *Staphylococcus aureus* (*S. aureus*) (NCTC 6571) and *Escherichia coli* (*E. coli*) (ATCC 25922).

## 2. Materials and Methods

### 2.1. Chemicals and Standards

Optima^®^ LC–MS grade water, acetonitrile (ACN) and methanol (MeOH) were supplied from Thermo Fisher Scientific (Waltham, MA, USA). Trifluoroacetic acid (TFA) was purchased by Romil Ltd. (Cambridge, UK). Formic acid (FA), dichloromethane (DCM), tris (hydroxymethyl) aminomethane (Tris), pepsin from porcine gastric mucosa (P6887), and pancreatin from porcine pancreas (P7545), α-chymotrypsin from bovine pancreas (CHY5S), TSB (tryptic soy broth) and TBX (tryptone bile X-glucuronide), sodium deoxycholate (SDC) were purchased from Sigma-Aldrich (St. Louis, MO, USA). The protease inhibitor cocktail was from Promega (Madison, WI, USA). Ultrapure water (resistivity 18.2 MΩ cm) was obtained by an Arium water purification system (Sartorius, Göttingen, Germany). Solid phase extraction (SPE) C18 cartridges (1 g 6 mL, Agilent Technologies, Santa Clara, CA, USA) were provided by BOND ELUT (Varian, Palo Alto, CA, USA). Cartridges packed with 500 mg Carbograph 4 were supplied from Lara S.R.L. (Lara S.r.l., Formello, Italy). Pierce bicinchoninic acid (BCA) Protein Assay Kit was purchased from Thermo Fisher Scientific (Waltham, MA, USA).

### 2.2. Fish Protein Extraction

Yellowfin tuna (*Thunnus albacares*) was purchased from a local fish market and the skin, bones and entrails were eliminated to prepare fillets, then aliquoted and stored at −20 °C. One-hundred grams of muscle sample was reduced to a fine powder after being exposed to liquid nitrogen with mortar and pestle. Then, 10 g of the powder were treated with 30 mL of cold lysis buffer consisting of 80 mmol of Tris-HCl (pH 8) with 2% SDC added with MSSAFE protease and inhibitor cocktail (Sigma-Aldrich, Milan, Italy), used according to the manufacturer’s instructions. The sample was extracted with 15 alternating cycles of 1 min of agitation on vortex and incubation on ice of 1 min for a total of 30 min, then centrifuged at 9400× *g* at 4 °C for 30 min to separate the cellular debris and finally transferred to a new test tube. Proteins were precipitated and collected as previously reported by adding 4 volumes of 10% (*w*/*v*) trichloroacetic acid in ice-cold acetone [23]. Proteins were quantified by the BCA assay using bovine serum albumin (BSA) as standard and kept at −80 °C until further use. The protein concentration was 64.81 ± 0.12 mg mL^−1^, corresponding to a protein recovery extraction of 12.96 ± 0.13%. These data are in agreement with the literature data [24].

### 2.3. Simulated In Vitro Gastrointestinal Digestion

One gram of proteins was solubilized in urea 8 mol L^−1^ (pH 8.8) in Tris-HCl 100 mmol L^−1^ and subjected to a simulated in vitro gastrointestinal digestion as previously described [25].

The obtained hydrolysate was treated and enriched with two different purification systems, namely graphitized carbon black (GCB) and C18 cartridges. For GCB purification, the hydrolysis was quenched by adding 5 mol L^−1^ HCl to reach pH 2; for the C18 purification step, TFA was employed for stopping the reaction.

### 2.4. Hydrolyzed Peptide Purification

#### 2.4.1. Medium-Sized Peptides Purification

Solid-phase extraction (SPE) on C18 was used to concentrate the medium-sized peptides from the fish digested, as previously described [26]. Briefly, the cartridges were preliminary washed with ACN and conditioned with 0.1% TFA; then, the fish extract was loaded and the column was washed again. Elution was performed with ACN/H_2_O (50:50, *v*/*v*) containing 0.1% TFA, and the eluates were dried in a SpeedVac SC250 Express (Thermo Savant, Holbrook, NY, USA). The residue was reconstituted in 100 μL of 0.1% formic acid in H_2_O for the subsequent chromatographic analysis. Six dried aliquots were stored at −80 °C until the antimicrobial bioactivity test.

#### 2.4.2. Short-Sized Peptide Purification

Short-sized peptide purification was obtained thanks to a specific protocol which was previously optimized in our previous work on a GCB sorbent [21]. After washing to remove the impurities from the cartridge, the GCB sorbent was first activated with 0.1 mol L^−1^ HCl and then conditioned with 20 mmol L^−1^ TFA. Protein digest was then loaded and the cartridge was again washed. Finally, peptides were eluted with DCM/MeOH 80:20 (*v*/*v*) with 20 mmol L^−1^ TFA by back flushing elution. Eluates were dried up at room temperature in a Speed-Vac and the residue reconstituted in 200 μL water for the subsequent RP separation. Six dried aliquots were stored at −80 °C, until the antimicrobial bioactivity test.

### 2.5. Chromatography–Mass Spectrometry Analysis of Peptide Samples

#### 2.5.1. Analysis of Medium-Sized Peptides by NanoHPLC–MS/MS

Medium-sized peptides were analyzed by nanoHPLC on an Ultimate 3000 (Thermo Fisher, Scientific, Bremen, Germany) coupled to an Orbitrap Elite mass spectrometer (Thermo Scientific, Bremen, Germany) [27]. Twenty microliters were injected and preconcentrated on a μ-precolumn (Thermo Fischer Scientific, 300 μm i.d. × 5 mm Acclaim PepMap 100 C18, 5 μm particle size, 100 Å pore size) at 10 μL min^−1^ flow rate of a premixed mobile phase H_2_O/ACN 98:2 (*v*/*v*) containing 0.1% (*v*/*v*) TFA. Then, the samples were separated on an EASY-Spray column (Thermo Fischer Scientific, 15 cm × 75 μm i.d. PepMap C18, 3 μm particles, 100 Å pore size) operated at 250 nL min^−1^ and at 20 °C. Peptide spectra were acquired in the 380–1800 *m*/*z* range at 30,000 resolution for the full scan. MS/MS spectra were acquired at 15,000 resolution in top 10 data-dependent acquisition (DDA) mode with the rejection of singly charged ions and of unassigned charge states. Precursors were fragmented by higher-energy collisional dissociation (HCD) with 35% normalized collision energy and a 2 *m*/*z* isolation window. For each sample, three technical replicates were performed. Raw data files were acquired by Xcalibur software (version 2.2, Thermo Fisher Scientific, Bremen, Germany).

#### 2.5.2. Analysis of Short-Sized Peptides by UHPLC–MS/MS

Samples were analyzed by RP chromatography as previously described [19] on a UHPLC Vanquish binary pump H coupled to a Q Exactive mass spectrometer (Thermo Fisher Scientific, Bremen, Germany) by a heated electrospray (ESI) source. Samples were injected onto a Kinetex XB-C18 (100 × 2.1 mm, 2.6 μm particle size, Phenomenex, Torrance, CA, USA) operated at 40 °C. Full scan spectra were acquired in the positive ionization mode in the range of *m*/*z* 150–750 with a resolution (full width at half maximum, FWHM, *m*/*z* 200) of 70,000. HCD MS/MS spectra acquisition was performed using top 5 DDA mode at 35% normalized collision energy and 35,000 (FWHM, *m*/*z* 200) resolution. An inclusion list with the exact *m*/*z* values for unique singly charged precursor ions was used for the DDA (4980 unique masses). Inclusion lists were prepared using MatLab R2018, as previously described [20].

### 2.6. Qualitative Antimicrobial Assay

The microorganisms chosen to test the antibacterial activity were *E. coli* (ATCC 25922) and *S. aureus* (NCTC 6571). The standard bacteria were obtained from the Department of Pharmaceutical, Istituto Zooprofilattico del Mezzogiorno Portici, Italy. Bacterial cultures were stored on nutrient agar at a temperature of 4 °C. The starting cultures of the two bacteria were prepared as described in the Supplementary Materials word file. The bacterial concentration was analyzed by a densitometer with an optical density of 0.12 OD, wavelength 600 nm. The initial concentration of the two standard bacteria was 0.5 McFarland, corresponding to 1.5 × 10^8^ cfu mL^−1^. This concentration was diluted to a final concentration of 10^3^ cfu mL^−1^. The antibacterial assay was carried out as previously described with some modifications [28]. Briefly, the purified samples on C18 and GCB were solubilized in ultra-pure water to obtain the final concentrations of 7.0 mg mL^−1^. Bacteria were added to peptide extracts and incubated at 37 °C for 4 h. The bacterial growth was measured at 600 nm in order to select the bacterial strain for the quantitative antimicrobial assay.

### 2.7. Quantitative Antimicrobial Assay—Minimum Inhibiting Concentration (MIC) Determination

MIC was determined by the broth macrodilution method to determine the power of antimicrobial activity of the digested tuna towards Gram-positive bacteria [29,30]. Stock solutions of the digested tuna in deionized water were prepared as follows: extracts of C18 (7.0 mg mL^−1^ A280 1.606) and extracts of GCB (7.0 mg mL^−1^ A280 2.276) were diluted with tryptic soy broth (TSB Sigma-Aldrich) to working concentrations of 3.5 mg mL^−1^, 1 mg mL^−1^ and 500 mg mL^−1^, to obtain a final volume of 1 mL. Serial two-fold dilutions of the working concentrations were made in the tube using TSB (in triplicate). All controls including sterility control (TSB and deionized water), positive control (containing bacterial suspension and TSB, without antimicrobial substance), and the negative control (TSB and extracts of digested tuna) were also distributed in sterile tubes of 2 mL. Each test and the positive control tube were inoculated with 100 μL bacterial suspension with final concentrations of 1 × 10^3^. All experiments were performed in triplicate. In the first step, the tubes as prepared were incubated 37 °C for 4 h under constant agitation (EW-51900-19 Stuart Equipment, Stone, UK), then 10 μL of each tube were diffused to the Baird parker agar plate (ISO 6888-1). The optical density of the tube was measured at 620 nm (Eppendorf BioSpectrometer^®^ basic) and the bacterial growth on the Baird parker agar plate after 24 h and 48 h at 37 °C. For determining the minimum bactericidal concentration (MBC), the samples taken from each tube were spread on Mueller-Hinton agar plates and incubated overnight at 37 °C. MBC was considered the concentration which corresponded to no bacterial growth. Each of these tests was performed twice (*n* = 2).

### 2.8. Peptide Identification

#### 2.8.1. Medium-Sized Peptide Identification

Raw MS/MS data files were submitted to Proteome Discoverer software (version 1.3, Thermo Scientific, Bremen, Germany) and the Mascot (v.2.3.2, Matrix Science) search engine. The searches were performed against the UniProt database, restricted to proteins of the Eupercaria order (v3.80, 179,974 sequences, 5,358,736 amino acid sequences). The built-in decoy search option for Mascot search was enabled. Parameters for the database search were: digestion with no enzyme, peptide charges from +2 to +5, precursor mass tolerance of 10 ppm and fragment mass tolerance of 0.8 Da; acetylation (N-term), oxidation (M) and deamidation (N, Q) were used as dynamic modifications; no static modification was selected.

#### 2.8.2. Short Peptide Identification

The identification of short peptides in the GCB extracts was accomplished as described thanks to a dedicated data processing workflow implemented on Compound Discoverer (v. 3.1, Thermo Fisher Scientific, Bremen, Germany) [22]. Briefly, the workflow allowed to extract the masses from the raw data files according to customized parameters for predicting the composition tool, aligning them, removing the signals of the blank or lacking MS/MS spectrum and using the comprehensive short peptide lists to match the extracted features. Manual validation of the MS/MS spectra was also aided using the compound class scoring tool, which allowed to automatically match typical product ions deriving from amino acids at N-terminus, C-terminus and in the middle of the sequence, and assign them to 20 compound classes (one for each natural amino acid). The tentative identification of short peptides was achieved according to the diagnostic fragmentation spectra, aided by mMass, which allows performing the in silico fragmentation of peptides [31].

## 3. Results and Discussion

In this study, medium- and short-sized peptides were hydrolyzed and identified from yellowfin tuna samples. With the purpose of obtaining both classes of peptides, a simulated gastrointestinal digestion was carried out, the ability of these enzyme mixture to generate small peptides and free amino acids as well is well known [32]. Together with the development of a platform able to characterize both type of peptides, an antimicrobial assay was carried out on the two obtained fractions. AMPs’ mechanisms of action against bacteria are the most intensively studied to date [8]. AMPs are able to protect the host against a broad range of Gram-positive and Gram-negative bacteria, mostly by direct antimicrobial action, attributed to their ability to form pores and disrupt cell membranes [33]. AMPs are expressed in different fish tissue [12,34], depending on the infective bacteria, and they can also display an antibacterial function against antibiotic-resistant bacteria strains. Moreover, their antimicrobial capacity is related to some peculiar characteristic, such as the amphipathic structure which is pivotal for the antibacterial activity [33]. Most AMPs are cationic, able to fold into amphiphilic α-helices and accumulate by several fold at the negatively charged surface of the Gram-negative or Gram-positive bacteria, leading to the surface destabilization and permeation. This way, AMPs can enter the bacterial cells.

### 3.1. Antimicrobial Activity of C18 and GCB Digested Fractions

The agar diffusion assay is commonly employed to test the antimicrobial activity of food antimicrobials, peptides or hydrolysates. The antibacterial activities of two enzymatic digested fractions were evaluated against Gram-positive (*S. aureus*) and Gram-negative (*E. coli*) bacteria. Preliminary results showed that purified digests on C18 and GCB possessed higher antibacterial activity on Gram-positive than on Gram-negative bacteria, therefore subsequent tests with longer incubation times were carried out only on *S. aureus*.

The MIC, namely the lowest concentration of peptide extracts at which no growth was observed after the incubation of the samples against the tested bacterial strain, was found to be 1.0 ± 0.1 mg mL^−1^ for the C18 extract and 3.5 ± 0.1 mg mL^−1^ for the GCB extract. The results are shown in Table 1.

These results were comparable to the values obtained in two previous works against *S. aureus* bacteria; the first paper was carried out on yellowfin tuna viscera bioactive peptide hydrolyzed fractions, where the MIC value was 0.5 mg mL^−1^ [35]; in the second one, the antimicrobial activity of the fractions isolated from bromelain hydrolysate of leatherjacket (*Meuchenia* sp.) insoluble muscle proteins showed a MIC value of 4.3 mg mL^−1^ [36].

In this work, we decided to not synthesize peptides. In fact, the MIC value sometimes could be lower for the single peptide rather than the whole mixture. A synergistic effect could in fact occur when several peptides are simultaneously present. This phenomenon has been reported for other matrices as well, and can lead to the preparation of a bioactive peptide fraction rather than the isolation of single peptides, whose cost would also be much higher. A recent study has also shown that the interactions of AMPs is mostly synergistic, and a combination of AMPs displayed stronger synergism than a single peptide. This could imply that synergism is a common phenomenon in AMP interaction [37].

### 3.2. Characterization of Hydrolyzed Medium-Sized Peptides in Fish

The identification of medium-sized peptides was achieved by using an established peptidomics approach. Currently, the use of techniques borrowed from shotgun proteomics represents a reference field for the identification of peptides in a complex sample, as digests. The challenges in this field for a confident and comprehensive peptide identification is provided by the limit in complete protein sequence databases. For most organisms, this is not an issue [38], while it still is in food characterization [39,40], as in the case of this work. A complete protein sequence database for tuna is currently not available at the time of this work, therefore proteins can be identified basically by similarity to the proteins reported for close organisms, to protein sequences obtained by translating transcriptomic data or by de novo approaches, which do not rely on protein sequence databases. In this work, a peptide identification by homology in the Thunnini taxonomy (1107 entries) was carried out, as it is the most convenient and less time consuming. The analysis of yellow fin tuna provided a total of 403 identified peptides (Table S1). The identified peptides were submitted to search in the BIOPEP [41] and PeptideDB databases, which contain a list of biologically active and validated peptide sequences, in order to determine if any already established bioactive peptide was present in the yellowfin tuna extracts. However, the search against the two databases did not furnish any match with the already found sequences.

**Figure 1:**
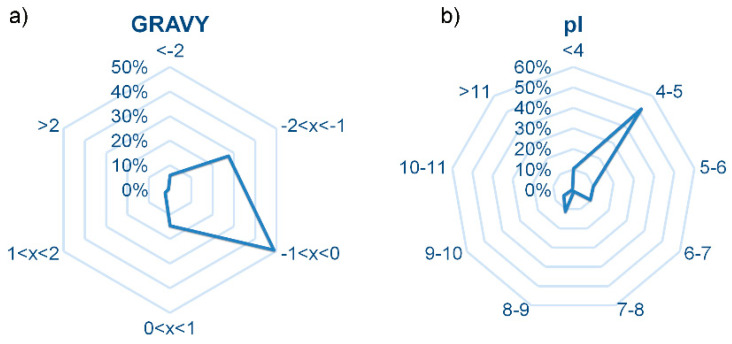
Distribution of grand average of hydropathy (GRAVY) (**a**) and isoelectric points (pI) (**b**) values for the identified medium-sized peptides.

The antimicrobial activity of a peptide is strictly entwined with their structure, with their molecular weight (1–5 kDa) and their net charge. In particular, most food-derived peptides possess low molecular weight with hydrophobic residues including Leu and Val in their N-terminus and Pro, His, Tyr, Trp, Met, and Lys in their sequences [42]. AMPs are classified as cationic or anionic on the basis of their net charge and their amino acidic residues; cationic peptides are rich in Arg, Lys or His and possess a net charge comprising between +2 and +9 while anionic peptides, first reported in the 1980s, are rich in aspartic and glutamic acid. Structural characterization shows anionic AMPs to generally range in a net charge from −1 to −7 and in length from 5 residues to about 70 residues [43]. The physicochemical features of the identified peptides were considered. Figure 1 describes the distributions of the identified peptides according to the calculated grand average of hydropathy (GRAVY) and isoelectric point (pI) values. Considering the GRAVY values, the peptides identified in this study were mostly hydrophilic, with negative values (82% of peptide hits), mostly with intermediate polarity (49% have a value between −1 and 0). As far as pI are0concerned, they were mostly <7 (82% of peptide hits). The pI values allowed to estimate that most of the identified sequences were anionic.

Finally, a prediction of the potential antimicrobial activity was also carried out with specific online available bioinformatic tools. It was demonstrated that the shorter length translates into easier manufacturing, (synthesis, modification, structure optimization) and cheaper production than that of long AMPs. In particular, short-length AMPs have been shown to have enhanced antimicrobial activities, higher stability, and lower toxicity for human cells [44]. A bioinformatic tool, Deep-AmPEP30, capable to evaluate the antimicrobial activity of such short peptides (5–30 amino acids long), has been presented, demonstrating its capability in predicting bioactive sequences from genomes. One sequence identified in this study proved experimentally more antimicrobial than ampicillin [44]. Deep-AmPEP30 was tested to score the bioactivity of the identified peptide sequences in tuna. From this analysis, 75 sequences scored above 0.5 and were potentially antimicrobial. For an increased confidence, only sequences with a score > 0.9 were considered, as described in the original work. This process reduced the bioactive candidate sequences to eight. All of them also had a RF-AmPEP30 score (which is also calculated by the same tool) above 0.5, which further supports the likability of them been potentially antimicrobial. To further investigate these results, CPPpred [45] was used to predict which sequences could also be cell penetrating. The scores were in the range of 0.27–0.69. Two sequences scored above 0.6 (KKLGELLK and KLGELLK) and could be good candidates for the validation of their activity in vitro.

### 3.3. Characterization of Hydrolyzed Short-Sized Peptides in Fish

By means of untargeted suspect screening approach, 572 potentially bioactive short-sized peptides were identified (Table S2), which is, to the best of our knowledge, the higher number of short peptides ever reported in fish samples. To date, most of the works were focused on longer amino acidic sequences, but the recent discovery of several short AMPs possessing potent inhibitory activity against bacteria with high specificity and low toxicity to human cells have led to an exciting turning point in short AMP research [46].

Until now, for a lack of dedicated analytical methodologies specifically dedicated to enrichment, the purification and identification of short peptides, scarce literature was produced in this regard. However, already in 1992 synthetic di-peptides containing only Leu and Lys units were reported to be active against both Gram-negative and Gram-positive bacterial strains [47]; tri-peptides containing Leu, Lys and Trp residues have also been evaluated for antimicrobial activity after de novo identification [48]. In the BIOPEP database, 2 short AMPs are already presented and validated in milk samples [19,20]. Moreover, some recent studies have demonstrated that shorter peptides possess different advantages in preparation, less toxicity, and stability [49]. Their recovery from food or by-product matrices could be cost-effective with respect to the chemical synthesis which is an expensive process.

As is possible to see from the list provided in Table S2, most of these sequences possess the typical amino acidic residues which were already found in short AMPs.

**Figure 2:**
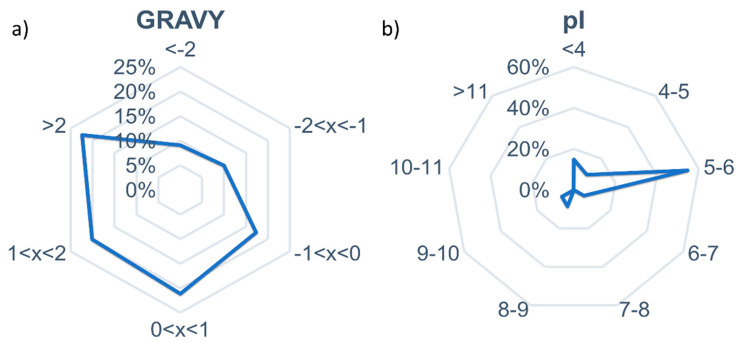
Distribution of the GRAVY (**a**) and pI (**b**) values for the identified short peptides.

In order to understand the short peptides’ physico-chemical properties, the GRAVY index and pI were calculated, and the results are shown in Figure 2.

As far as the GRAVY values are concerned, most of the identified short peptides in this study were hydrophobic, with a percentage around 63% of the total identified peptides with a positive calculated GRAVY index. This result, compared with the GRAVY index for medium-sized peptides, highlighted that the use of a mixture of pepsin and pancreatin was able to generate more hydrophobic and aromatic short peptides, containing for instance Leu, Ile, Phe and Tyr. This behavior was already demonstrated in pigeon pea digest [50,51].

Regarding pI, most peptides possessed a value comprising of between 5 and 6, therefore resulting less anionic in comparison with medium-sized peptides. These data could justify the results obtained in an antimicrobial assay in which the C18 purified extract exhibited a higher activity with respect to the GCB extract (see Table 1). A number of studies have shown that anionic peptides in fact display high antimicrobial efficacy with low MICs when directed against a wide range of Gram-positive bacteria [52], confirming our data in which the antimicrobial activity was higher on *S. aureus*.

## 4. Conclusions

The discovery of new bioactive peptides can play a crucial role in human health research, thanks to the many possibilities of designing new functional nutraceuticals and/or pharmaceuticals. In particular, fish-derived bioactive AMPs could be the new source of novel antimicrobial drugs in the future. In fact, they could serve as vaccines for inactive specific pathogens, and they could also be used in food preservatives and supplements. Therefore, the development of innovative analytical strategies for discovering new antimicrobial substances is very important, especially since the number of pathogenic bacteria that are resistant to conventional antibiotics is rising. Further studies should therefore be carried out for exploring the effects of this peptide on the human body. Moreover, this work was the first in which short peptides were also considered and identified, highlighting their antimicrobial potential. More studies should be carried out in order to confirm the potentiality of these short amino acidic sequences as possible therapeutics.

## Figures and Tables

**Table 1:** Minimum inhibitory concentration (MIC) and optical density measured at a wavelength of 600 nm (OD600) for the various dilutions of the extracts incubated with *Staphylococcus aureus.*

	C18 Extract		GCB Extract	
Concentration (mg mL^−1^)	MIC (cfu mL^−1^)	OD600	MIC (cfu mL^−1^)	OD600
7.0	0	0.000	0	0.000
3.5	0	0.000	0	0.000
1.0	0	0.000	1	0.021
0.5	3	0.018	3	0.023

## Supplementary Materials

The following are available online at https://www.mdpi.com/2304-8158/9/9/1185/s1, Supplementary Materials word file: Preparation of two bacterial strains; Table S1: List of medium-sized identified peptides; Table S2: List of short identified peptides.
