# Supplementary material for: Identification and Antimicrobial Activity of Medium-Sized and Short Peptides from Yellowfin Tuna (Thunnus albacares) Simulated Gastrointestinal Digestion
Source: Foods. 2020 Aug 27;9(9):1185. doi: 10.3390/foods9091185 (PMC7555217; doi:10.3390/foods9091185)
Supplement: Supplementary file 1 [file foods-09-01185-s001.pdf]

Supplementary Material

Identification and antimicrobial activity of medium-sized and short peptides from yellowfin tuna (*Thunnus albacares*) simulated gastrointestinal digestion

Andrea Cerrato[1], Anna Laura Capriotti[1], Federico Capuano[2], Chiara Cavaliere[1], Angela Michela Immacolata Montone[2,3], Carmela Maria Montone[1*], Susy Piovesana[1], Riccardo Zenezini Chiozzi[4], and Aldo Laganà[1,5]

[1]Department of Chemistry, Università di Roma "La Sapienza", Piazzale Aldo Moro 5, 00185 Rome, Italy

[2]Istituto Zooprofilattico Sperimentale del Mezzogiorno, Via Salute 2 Portici (NA)

[3]Department of Industrial Engineering, Università degli Studi di Salerno, Via Giovanni Paolo II, 132 I-84084 Fisciano (SA), Italy

[4]Biomolecular Mass Spectrometry and Proteomics, Bijvoet Center for Biomolecular Research, Utrecht Institute for Pharmaceutical Sciences, Utrecht University, Padualaan 8, Utrecht, 3584 CH, Netherlands

[5]CNR NANOTEC, Campus Ecotekne, University of Salento, Via Monteroni, 73100 Lecce, Italy

*Corresponding author: Carmela Maria Montone

Department of Chemistry

Università di Roma "La Sapienza"

Piazzale Aldo Moro 5

00185 Rome, Italy

E-mail: carmela.maria@uniroma1.it

tel: +39 06 4991 3062

The ATCC bacterial strains are stored inside Microbank TM at -20 °C:

1. Defrost at room temperature, paying particular attention to the safety standards provided by class 2 laboratories
2. With a sterile loop, take a small sphere from the microbank and perform the microbiological smear on blood agar plates and incubate at 37 ° for 24 hours *
3. Remove bacteria colonies from the bacteria plate and inoculate the tube containing the Mueller-Hinton broth 10 ml, maintain this bacterial stock culture exponentially, incubating for about 4 hours at 37 °
4. Use a densitometer McFarland DEN-1 (0.3 - 15.0 unit) for obtened 0.5 unit of McFarland that corresponds roughly to 10^8 bacteria/mL , or It Is possible use a McFarland standard that corresponds to a suspension of BaC12 prepared in H2S04 [0.05 mL of 1% BaC12 (w/v) in 9.95 mL of 10% H2S04 (v/v)] and compare the inoculum visually
5. Adjust the suspension to 10^3 bacteria/mL (a dilution to 1 : 10)

In order to verify the antimicrobial activity of the two extracts against the *Staphylococcus aureus bacteria strain, two antibiotics were used as controls: Ampicillin (25* µg/mL) *and Polymyxin B (400*µg/mL). Data are shown in Table

Antibiotics and extracts	Bacteria strain	UFC/mL inoculo	MIC mg ml^{-1}	UFC 24 h incubation	MCB mg ml^{-1}
Ampicillin	*S.aureus*	1,5 x 10^3	1.5 X 10^{-6}	1,0 x 10^{-1}	1.5 X 10^{-6}
Polymixin B			2,4 x 10^{-5}	3.7 x 10^{-2}	2,4 x 10^{-5}
C18			10,0 x 10^{-1}	0	10,0 x 10^{-1}
GCB			35 x 10^{-1}	0	35 x 10^{-1}
